# Integrative Medicine as a Vital Component of Patient Care

**DOI:** 10.7759/cureus.3098

**Published:** 2018-08-04

**Authors:** Richard Gannotta, Shaista Malik, Alvin Y Chan, Kamran Urgun, Frank Hsu, Sumeet Vadera

**Affiliations:** 1 Chief Executive Officer, University of California, Irvine, Orange, USA; 2 Integrative Medicine, University of California, Irvine, Irvine, USA; 3 Neurological Surgery, University of California, Irvine, Orange, USA; 4 Neurological Surgery, University of California Irvine, Orange, USA; 5 Neurosurgery, University of California, Irvine, USA

**Keywords:** alternative medicine, alternative and complementary medicine

## Abstract

The landscape of medicine in the United States has been slowly progressing toward a more holistic and individualized approach to healing. Part of this progress has been the integration between western and alternative forms of medicine, a concept that has been described as “integrative medicine.” This approach to healthcare incorporates a patient’s mind, spirituality, and sense of community into the healing process. Integrative medicine has been typically well received and the demand has been steadily increasing in primary US hospitals. Here we cover a number of topics that include the definition of integrative medicine, its potential benefits, current examples of successful implementations, and potential barriers to its expansion. The aim was to give a primary on integrative medicine and its current state for healthcare providers.

## Introduction and background

Culturally diverse communities may benefit from an integration between western and alternative forms of medicine that takes a holistic approach to caring for patients. The concept has been given the term “integrative medicine,” which goes beyond simply combining different forms of medicine and allows for an individualistic approach to patient care that incorporates the mind, spirituality, and sense of community as well as the body. This benefit has been shown in a variety of settings across different nations. For example, there was a case-controlled study conducted in South Korea that compared western-only with integrative medicine (i.e., an individualistic combination of western and traditional Korean medicine) in managing patients with acute strokes [1]. The results were that although patients who received integrative medicine incurred slightly higher costs than those who underwent only western medicine but that they also had a reduced all-risk mortality at both three and 12 months after discharge, which may have prevented additional costs from future admissions. Similarly, another retrospective study in Texas showed that patients who received integrative medicine were more likely to have reduced pain that resulted in an approximately 4% decrease in hospital costs [2]. These findings have implications for care providers and stakeholders, as research has shown that integrative medicine likely improves patient care and outcomes while ultimately reducing hospital costs.

Here we review the current evidence for integrative medicine in the United States as well as discuss potential benefits and barriers to implementation. The aim was to abate the stigma associated with the field and educate readers about the potential it has for improving patient care. Most patients who seek out integrative medicine alternatives are self-referred [3]; therefore, educating practitioners on potential integrative options would likely benefit patient care and increase access.

## Review

What is integrative medicine?

Integrative medicine is the treatment of patients through spiritual, emotional, mental, and environmental in addition to the physical means. The general principle is that all aspects of the patient are considered in treating illness, which include natural and less invasive alternatives when possible. The patient and health care provider form a partnership that allows for a holistic approach that includes beliefs, overall wellness, and community to the healing of the mind and body. Specific techniques can include acupuncture, nutritional advisement, mind-body therapies, and holistic massages [4], but may involve any effective natural treatment outside of conventional methodology. However, the treatments are still rooted in scientific discovery and inquiry [5]. Indeed, research and scientific investigation of efficacy are prevalent and an important guide of treatment [3]. The way integrative medicine is provided can vary by location but it is ubiquitous across different avenues of healthcare. Many integrative medicine centers are associated with major teaching hospitals or healthcare organizations and are often available as consultation, comprehensive care, or a primary service [3].

Benefits of integrative medicine

The demand for integrative medicine in primary US hospitals has been increasing. To meet this demand, a number of hospitals have been trialing integrative medicine programs and investigating the result on patient care. A number of studies and reviews have detailed that these types of programs have been well received with positive outcomes [6-10]. When an integrative medicine clinic was implemented in a large California community hospital, 160 patients were recruited to investigate the effects of the clinic on their overall health [11]. The researchers concluded that clients reported substantial improvements in their symptoms and that an integrative health clinic could co-exist with a traditional major western medical center. The main limitation was that the trial was not controlled and thus they could not make any definitive statements about the outcomes.

Another benefit is symptom improvement during the course of disease, which is especially true within the realm of cancer when conventional treatments are often limited [4, 10, 12-14]. For example, a large portion of patients with breast cancer seeks alternative treatment in addition to conventional western medicine for symptomatic treatment [15]. Similarly, in an observational study of 238 unspecified cancer patients, who were referred to an inpatient integrative medicine consultation service, the patients were less likely to be distressed by the disease and more involved in their own care afterward [16]. The authors argue that the integrative medicine service helped the patients address their concerns, which helped with coping. Another case-controlled study of almost 500 patients with breast or gynecological cancer showed those undergoing integrative medicine were more likely to have an improvement in appetite, fatigue, cognitive and emotional functioning, pain, anxiety, sleep, and overall global health [17]. The benefits of integrative medicine are unsurprising. The concept is that bio-medicine does not necessarily address the complexity of healing or palliative treatment necessary to fully abate cancer symptoms, which can include emotional and spiritual distress beyond the physical pain. Also, cancer is not the only disease where integrative medicine has been successful but it has been an important topic of research.

Providing integrative medicine treatment plans may also increase patient satisfaction. The University of Michigan introduced an integrative medicine clinic in 2003 and investigated the effect by surveying 85 patients about their experience [18]. The program included shared decision making and an emphasis on understanding patient life stories to better put their illnesses into context during treatment. The results of the survey suggested that patients were satisfied with their experience at the clinic and that customizing integrative medicine regimens allowed to improvements in mental and emotional well-being in addition to symptomatic and disease improvement. Despite the limitations of the study, which included the voluntary nature of the surveys, the authors argued that the results were promising and should foster additional discussion about the potential benefits of integrative medicine clinics. It is intuitive that patients who feel more control over their treatment and believe they have more options are likely to be more satisfied.

There are potential drawbacks to integrative medicine. One is that treating multiple systems and modalities of the patient often requires multiple teams that may disagree on conflicting treatment avenues. This can be avoided likely if there is one main team who consults other specialists for different patient needs. In general, the positives outweigh the negatives and integrative medicine should be considered when appropriate.

Barriers to implementation

There are a number of barriers to implementing integrative medicine in modern western hospitals. First, there are increased hospital costs of adding programs and centers. At our institution, the University of California, Irvine, Medical Center, the Susan Samueli Center for Integrative Medicine was started due to a generous donation from Henry and Susan Samueli. We understand that donations are not always possible for starting these programs. However, there is evidence that integrative medicine programs may actually ultimately decrease hospitals costs in the long-term perspective due to decreased treatment requirements and fewer follow-up [7]. The patient population that is most likely to benefit immediately is the chronically ill and those suffering from depression [7]. Therefore, implementing integrative medicine programs slowly and then expanding as resources become more readily available is likely the most realistic course.

Second, integrative medicine programs may not be well received or practiced by clinicians, which could be due to ideological differences or poor education on the topic. An 18-question survey conducted on pediatric resident physicians at Baylor University in Texas showed that although over 80% of their patients had asked about integrative medicine options, almost 90% of the respondents felt their knowledge of integrative medicine needed expansion [19]. The authors concluded that lack of knowledge was the primary barrier to implementation in the resident physicians’ practices. There are a variety of ways to circumvent this barrier. The simplest way would be for physicians and other caretakers to undergo training in terms of their patients’ integrative medicine options [20]. For example, the University of Arizona Center for Integrative Medicine provides an online course for resident physicians to expand their knowledge on the subject [21]. Indeed, online courses for physicians have been shown to be effective education for teaching integrative medicine [22]. More formal education is also available and could be sought as well [23]. A similar alternative would be to implement integrative medicine as a core part of medical school curriculums [24]. Other methods include the development of a consulting integrative team or referral to an integrative medicine clinic.

There are likely a number of barriers that must be overcome for integrative medicine to be integrated into traditional western hospital. There may also be a hostility toward alternative medicines as well as cultural differences [15]. However, implementation of integrative medicine should be considered when possible due to its potential benefit for patients. Moreover, patients may also exhibit higher satisfaction with integrative treatment plans [18], which could lead to benefits for the providers and hospital stakeholders as well. Indeed, there is evidence that healthcare professionals are more likely than the general public to use integrative medicine techniques, which could be due to easier access or paradoxically more knowledge about its efficacy [25].

## Conclusions

The increasing popularity of integrative medicine is rooted in the effort to improve patient care and reduce suffering. The basis of its ideal is that true healing requires nurturing of the mind and soul in addition to the body, which goes above the idea of simply combining alternative with conventional medicine. A variety of therapies outside of conventional bio-medicine can often help patients deal with difficult illness. Integrative medicine services can take the form of a consultation, stand-alone clinic, or primary service. The benefits are likely reduced patient distress and pain in addition to lowering associated hospital costs. However, there are a number of barriers to implementation where the services are not already available, which include costs and low caregiver knowledge on integrative medicine options available. If these barriers can be overcome, patient care is likely to improve and that is the ultimate goal.
